# Large Screening Identifies ACE2 Positively Correlates With NF-κB Signaling Activity and Targeting NF-κB Signaling Drugs Suppress ACE2 Levels

**DOI:** 10.3389/fphar.2021.771555

**Published:** 2021-11-19

**Authors:** Meichen Yan, Yuan Dong, Xuena Bo, Yong Cheng, Jinbo Cheng

**Affiliations:** ^1^ Center on Translational Neuroscience, College of Life and Environmental Science, Minzu University of China, Beijing, China; ^2^ Department of Biochemistry, Medical College, Qingdao University, Qingdao, China

**Keywords:** angiotensin-converting enzyme 2, interferon-stimulated gene, NF-κB signaling, inflammatory cytokine storm, clinical drugs

## Abstract

Coronaviruses SARS-CoV-2 infected more than 156 million people and caused over 3 million death in the whole world, therefore a better understanding of the underlying pathogenic mechanism and the searching for more effective treatments were urgently needed. Angiotensin-converting enzyme 2 (ACE2) was the receptor for SARS-CoV-2 infection. In this study, we found that ACE2 was an interferon-stimulated gene (ISG) in human cell lines. By performing an ISG library screening, we found that ACE2 levels were positively regulated by multiple ISGs. Interestingly, ACE2 levels were highly correlated with ISGs-induced NF-κB activities, but not IFNβ levels. Furthermore, using an approved clinical durgs library, we found two clinical drugs, Cepharanthine and Glucosamine, significantly inhibited ACE2 level, IFNβ level, and NF-κB signaling downstream TNFα and IL6 levels. Our finding suggested the possible inhibitory effects of Cepharanthine and Glucosamine during SARS-CoV-2 infection and the subsequent inflammatory cytokine storm.

## Introduction

The pathogenic coronaviruses including SARS, MERS and new SARS-CoV-2 are cross-species transmitted from animal to human. SARS-CoV-2 infection not only causes severe respiratory syndrome, recent studies showed that SARS-CoV-2 infection also affects central nervous system (Abboud et al., 2020; Iadecola et al., 2020; Massad et al., 2020; Teng et al., 2020; Wang et al., 2021). Currently, due to the high transmission level, SARS-CoV-2 have infected over 156 million people and caused over 3 million death globally. Despite the availability of various vaccines for SARS-CoV-2, the recent emerging of new mutations of coronaviruses SARS-CoV-2 potentially impact the spread of this epidemic (Azgari et al., 2021; Chen et al., 2021; Di Giacomo et al., 2021; Jangra et al., 2021; Lubinski et al., 2021). Therefore, a better understanding of the underlying pathogenic mechanism, and the searching for more effective treatments including inhibition of coronaviruses infection and replication in the clinic are urgently needed.

Angiotensin-converting enzyme 2 (ACE2) acts as the main receptor for the infection of coronaviruses (Li et al., 2003; Garcia-Del-Barco et al., 2021). Multiple evidences reveals the increased level of ACE2 in COVID-19 patients (Zhuang et al., 2020; Pinto et al., 2020; Liu et al., 2021). Meanwhile, reduced risk of severe COVID-19 symptom is found associated with patients that has been prescribed with ACE inhibitor drugs (Hippisley-Cox et al., 2020), indicating the possible protective effect of ACE2 activity inhibition during the clinical treatment of COVID-19. Recently, ACE2 is reported as an interferon-stimulated gene (ISG) in human. Its expression is inducible by *in vivo* viral infection or *in vitro* interferon treatments (Ziegler et al., 2020). Upon bacteria or virus infection, interferon production is instantly induced, which subsequently causes the ISGs production. Meanwhile, the positive feedback of various ISGs in the regulation of interferon production is essential for the quick immune response and virus clearance (Levy et al., 2011; Kim et al., 1997). Moreover, innate immunity receptors, such as mitochondrial antiviral signaling protein (MAVS, also referred to as IPS-1, VISA or Cardif), and Cyclic GMP-AMP Synthase (cGAS), are also identified as ISG and participate in the quick interferon response (Ma et al., 2015; Cheng et al., 2016). In our previous study, we find that mitochondrial calcium uniporter protein (MCU)-mediated endoplasmic reticulum (ER) stress is involved in ISG response. Specifically, tumor necrosis factor receptor 1 (TNFR1), an ISG, is important for interferon production (Cheng et al., 2016). However, given the tight association between COVID-19 infection, ACE2 activity and the resulting inflammatory response, it is unclear whether the expression of ACE2 is regulated by ISGs. In severe COVID-19 patients, high level of inflammatory cytokine production, also referred as inflammatory cytokine storm, aggravates the symptom of infection and even contributes to the death of patients (Fara et al., 2020; Soy et al., 2020; Rabaan et al., 2021; Yang et al., 2021). Therefore, the relationship between ACE2 expression and inflammatory cytokine production caused by COVID-19 infection is importance for the clinical studies of COVID-19.

In this study, we found that ACE2 was an ISG in human cell lines. Through ISGs library screening, we found that several ISGs positively regulated ACE2 levels. Interestingly, we also found that ACE2 levels were significantly correlated with ISGs-induced NF-κB activities, but not IFNβ levels. Furthermore, through clinical-approved durgs library screening, we found two clinical drugs, Cepharanthine and Glucosamine, significantly inhibited ACE2 levels, IFNβ levels, and NF-κB signaling downstream TNFα levels and IL6 levels.

## Results

### Human ACE2 is an ISG in Human Cell Lines

**FIGURE 1:**
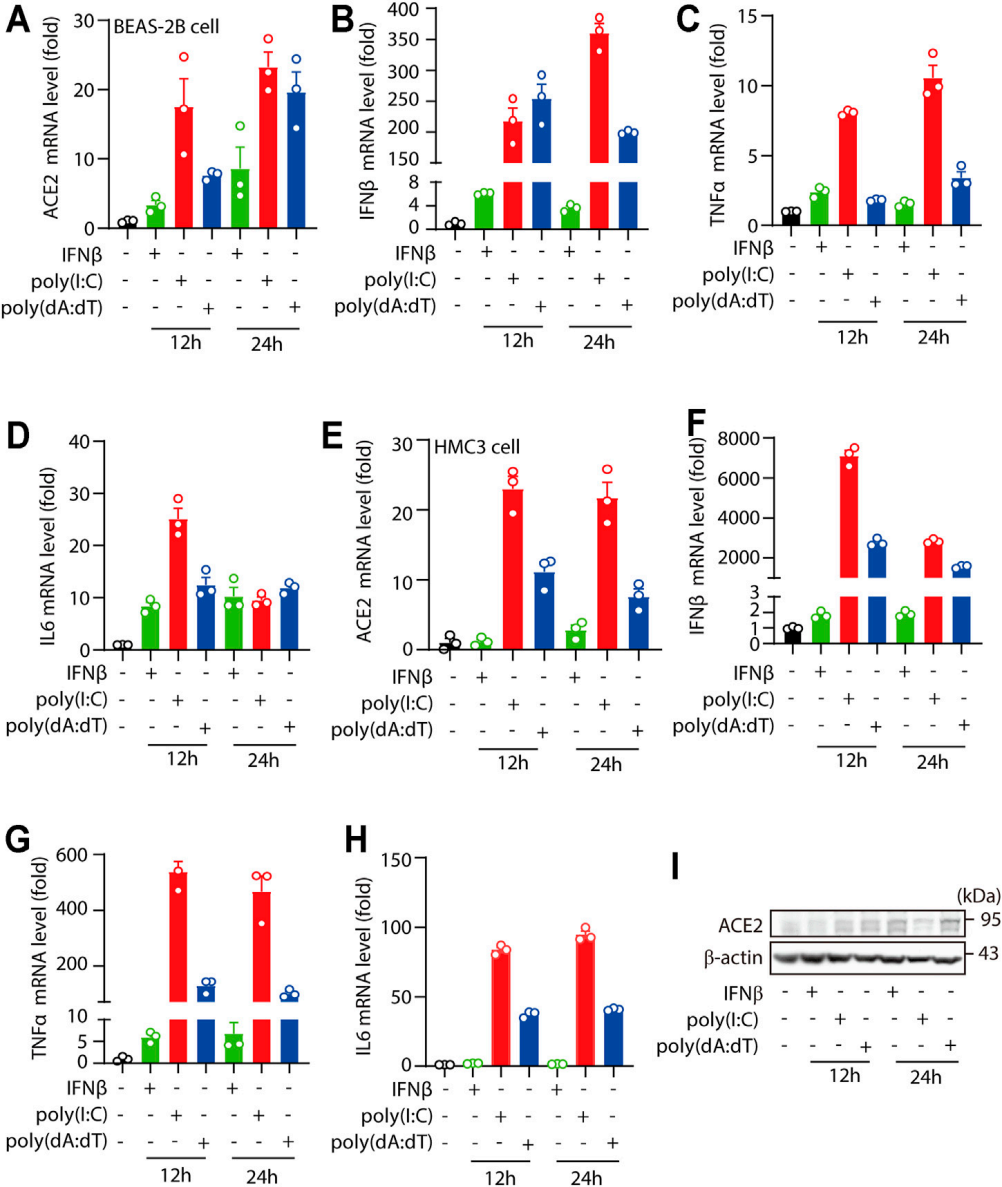
Human ACE2 is an ISG in human cell lines. **(A–D)** RT-PCR analysis of the expression of ACE2, IFNβ, TNFα and IL6 in BEAS-2B cells treated with IFNβ, transfected poly(I:C) or transfected poly(dA:dT) (complexed with Lipofectamine™ 2000 at a ratio of 1: 1 with the concentration of 1 μg/ml). **(E–H)** RT-PCR analysis of the expression of ACE2, IFNβ, TNFα and IL6 in HMC3 cells treated with IFNβ, transfected poly(I:C) or transfected poly(dA:dT) (complexed with Lipofectamine™ 2000 at a ratio of 1: 1 with the concentration of 1 μg/ml). **(I)** Immunoblotting analysis the levels of ACE2 levels in BEAS-2B cells treated with IFNβ, transfected poly(I:C) or transfected poly(dA:dT) for 24 h. Data were displayed as mean ± SEM. Experiments were carried out in triplicate, and at least three independent times.

For the *in vitro* characteristic study of human ACE2, human cell lines BEAS-2B cells and HMC3 cells were stimulated with IFNβ, viral RNA mimic poly(I:C) or viral DNA mimic poly(dA:dT) as indicated. We found that treatments of IFNβ, viral RNA mimic poly(I:C) and viral DNA mimic poly(dA:dT) signifcantly increased ACE2 mRNA levels in BEAS-2B cells (Figure 1A), along with significant enhancement of IFNβ, TNFα and IL6 levels (Figures 1B–D). Consistently, treatments of IFNβ, viral RNA mimic poly(I:C) and viral DNA mimic poly(dA:dT) in HMC3 cells also signifcantly increased the mRNA levels of ACE2, IFNβ, TNFα and IL6 (Figures 1E–H). Furthermore, we found that the protein levels of ACE2 were also increased upon IFNβ, viral RNA mimic poly(I:C) and viral DNA mimic poly(dA:dT) treatment (Figure 1I). Together, these results suggested that human ACE2 was an ISG in human cell lines.

### ACE2 Expression is Regulated by ISGs

**FIGURE 2:**
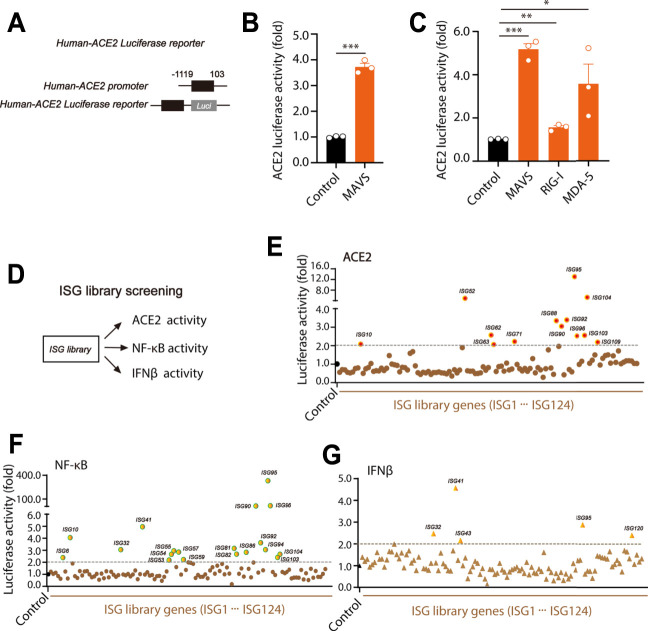
ISG library screens the effect of ISGs on human ACE2 luciferase activity. **(A)** Construction of human ACE2 luciferase reporter plasmids as indicated. **(B)** The plasmids of MAVS and human-ACE2 luciferase reporter 1 were transfected into HEK293T cells. 24  h after transfection, cells were lysed and the activity of human-ACE2 luciferase reporter was mearsured. **(C)** The MAVS signaling key components including MAVS, RIG-1 and MDA-5 were respectively transfected with human-ACE2 luciferase reporter into HEK293T cells. The luciferase activity of ACE2 was tested 24 h after transfection as indicated. **(D)** The model for the effects of ISGs on ACE2, NF-κB and IFNβ activities. **(E–G)** Single ISG from the ISG library was transfected into HEK293T cells together with ACE2 luciferase reporter, NF-κB luciferase reporter or IFNβ luciferase reporter, and individual luciferase activity was mearsured at 24 h after transfection. All the values were nomolized with the levels in control groups. Data were displayed as mean ± SEM. Experiments were carried out in triplicate with three independent times. *p < 0.05, **p < 0.01, ***p < 0.001.

Luciferase reporter system was constructed using human ACE2 promoter (−1119 to 103) and pGL3-luciferase reporter vector (Figure 2A). We found that overexpression of MAVS caused significant elevation in ACE2 luciferase activities (Figure 2B), suggesting the promotive effect of MAVS signaling in ACE2 expression. To further confirm the role of RLR signaling on ACE2 expression, key components MAVS signaling: retinoic acid inducible gene-I (RIG-I) and melanoma differentiation associated gene 5 (MDA-5) were overexpressed. In the results, we found that both RIG-I and MDA-5 significantly increased the level of ACE2 (Figure 2C).

In our previous study, through ISG library screening, we found TNFR1 positively regulated RLR signaling (Cheng et al., 2016). However, it was still unclear how ISGs regulate ACE2 levels and NF-κB activities. To further study the effect of ISGs on ACE2, IFNβ and NF-κB activities, an ectopic expression assay of a library containing 117 human ISGs was performed (Figure 2D). Each ISG was co-transfected into HEK293T cells with ACE2 luciferase reporter, IFNβ luciferase reporter or NF-κB luciferase reporter. In the results, we found that there were 13 ISGs (ISG10, ISG52, ISG62, ISG63, ISG71, ISG88, ISG90, ISG92, ISG95, ISG96, ISG103, ISG104 and ISG109) significantly promoted the expression of ACE2 (>2-fold). Notably, the highest ACE2 level was induced by ISG95 expression (Figure 2E). Furthermore, we found that there were 19 ISGs (ISG6, ISG10, ISG32, ISG41, ISG53, ISG54, ISG55, ISG57, ISG59, ISG81, ISG82, ISG86, ISG90, ISG92, ISG94, ISG95, ISG96, ISG9103 and ISG104) significantly increased NF-κB activities (>2-fold) (Figure 2F). Meanwhile, as shown in Figure 2G, only ISG32, ISG41, ISG43, ISG95 and ISG120 were found to increase IFNβ levels (>2-fold) (Cheng et al., 2016). There results suggested that the expression of ACE2, IFNβ and NF-κB were regulated by different ISGs.

### Human ACE2 Level Positively Correlates With NF-κB Activities

**FIGURE 3:**
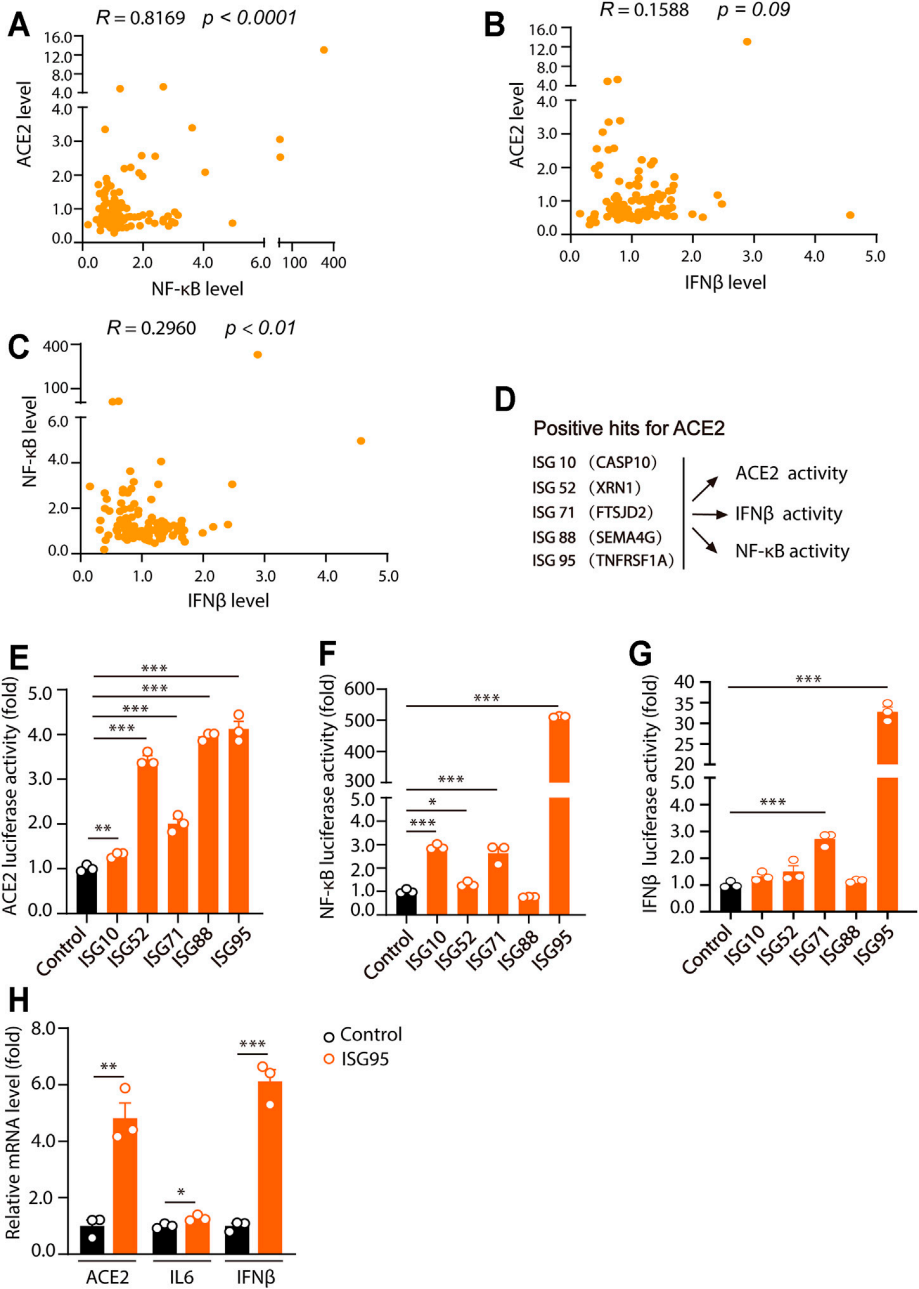
Human ACE2 levels are positively related with NF-κB activities. **(A)** The relationship between the effects of ISGs on ACE2 activities and NF-κB activities. **(B)** The relationship between the effects of ISGs on ACE2 activities and IFNβ activities. **(C)** The relationship between the effects of ISGs on NF-κB activities and IFNβ activities. **(D)** Model of the effects of five ISGs as indicated on ACE2, NF-κB and IFNβ luciferase activities. **(E–G)** HEK293T cells were transfected with five ISGs as indicated together with ACE2, NF-κB and IFNβ luciferase reporter. Cells were lysed 24 h after transfection and for luciferase assays. **(H)** BEAS-2B cells were plated into 12-well plate and cultured for overnight. 2 μg of ISG95 were transfected into BEAS-2B cells. 24 h later, the expression of ACE2, IL6 and IFNβ were analyzed. Data were displayed as mean ± SEM. Experiments were carried out in triplicate with three independent times. *p < 0.05, **p < 0.01, ***p < 0.001.

For further investigation of the effects of ISGs on ACE2, IFNβ and NF-κB levels, corrlation analysis was performed. Interestingly, we found that ACE2 levels were highly correlated with NF-κB activities (*R* value of 0.8169 and *p* value <0.0001) (Figure 3A), but not with IFNβ levels (*R* value of 0.1588 and *p* value of 0.09) (Figure 3B). Meanwhile, IFNβ levels were correlated with NF-κB activities (*R* value of 0.2960 and *p* value <0.01) (Figure 3C).

Among the ACE2-regulatory ISGs, ISG10 (CASP10), ISG52 (XRN1), ISG71 (FTSJD2), ISG88 (SEMA4G) and ISG95 (TNFR1) were further analyzed for their effects on ACE2, NF-κB and IFNβ levels (Figure 3D). In the results, we found that ISG10 (CASP10), ISG52 (XRN1), ISG71 (FTSJD2), ISG88 (SEMA4G) and ISG95 (TNFR1) all significantly increased ACE2 levels (Figure 3E). Meanwhile, ISG10 (CASP10), ISG52 (XRN1), ISG71 (FTSJD2), and ISG95 (TNFR1) caused significant increase of NF-κB level (Figure 3F). Meanwhile, only ISG71 (FTSJD2) and ISG95 (TNFR1) significantly increased the level of IFNβ (Figure 3G). Moreover, as overexpression of ISG95 increased the highest luciferase activities of ACE2, NF-kB and IFNβ, we examined the effects of overexpression of ISG95 on ACE2, IL6 and IFNβ levels in BEAS-2B cells. As shown in Figure 3H, overexpression of ISG95 significantly increased the mRNA levels of ACE2, IL6 and IFNβ in BEAS-2B cells. Together, these results suggested that ISGs-induced ACE2 levels were significantly correlated with NF-κB level, but not IFNβ level.

### Clinical Approved Drug Screening Shows Cepharanthine and Glucosamine Inhibit ACE2 Expression and NF-κB Signaling Downstream Cytokine Levels

**FIGURE 4:**
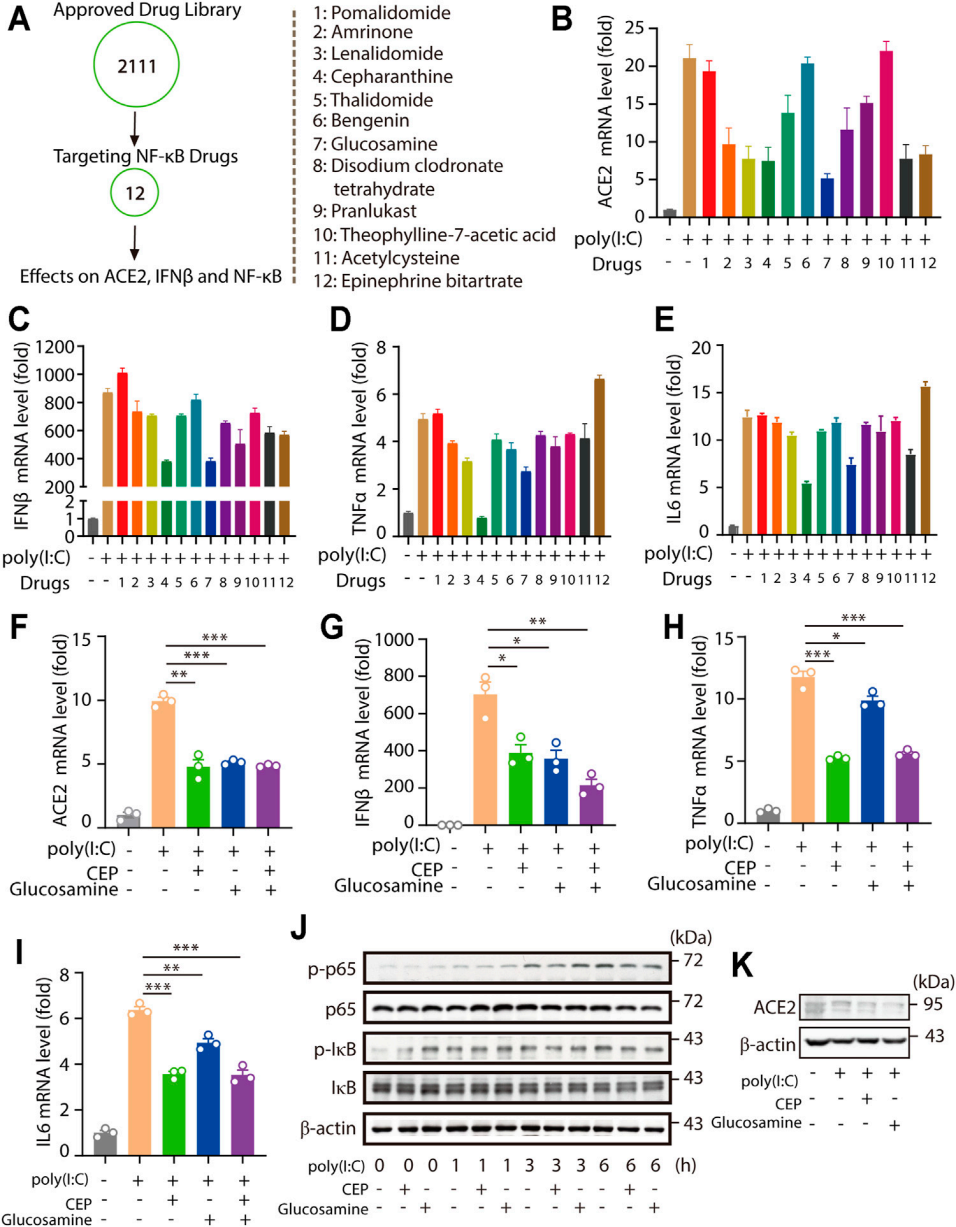
Drug library screening shows Cepharanthine and Glucosamine inhibit ACE2 expression and NF-κB signaling downstream cytokine levles. **(A–E)** RT-PCR analysis the expression of ACE2, IFNβ, TNFα and IL6 in HMC3 cells pretreated with 12 kinds of targeting TNF signaling drugs for 30 min, then treated with transfected poly(I:C) (1 μg/ml) for 24 h. All drugs were used at a concentration of 5 μM. **(F–I)** RT-PCR analysis the expression of ACE2, IFNβ, TNFα and IL6 in HMC3 cells pretreated with Cepharanthine (CEP) and Glucosamine for 30 min, then treated with transfected poly (I:C) (1 μg/ml) for 24 h. All drugs were used at a concentration of 5 μM. **(J)** Immunoblotting analysis the levels of p-p65, p65, *p*-IκB and IκB induced by poly(I:C) (1 μg/ml) for indicated time with or without Cepharanthine and Glucosamine treatment. **(K)** Immunoblotting analysis the levels of ACE2 induced by poly(I:C) (1 μg/ml) for 24 h with or without Cepharanthine and Glucosamine treatment. *p < 0.05, **p < 0.01, ***p < 0.001.

Multiple evidences showed that increased ACE2 levels and NF-κB downstream cytokine levels were presented in patients with severe COVID-19 symptom (Pinto et al., 2020; Soy et al., 2020; Zhuang et al., 2020; Rabaan et al., 2021). Together with our findings that ACE2 levels were highly correlated with NF-κB levels. Therefore, we hypothesized that treatment targeting NF-κB signaling potentially also produced inhibitory effects on ACE2 level, which was important for the prevention and clinical treatment of COVID-19 infection and the infection-caused inflammatory cytokine storm. 12 clinical approved drugs targeting NF-κB signaling were investigated for their effects on the levels of ACE2, IFNβ and NF-κB downstream cytokines (Figure 4A). Among these 12 approved clinical drugs, we found that Cepharanthine and Glucosamine significantly inhibited viral RNA mimic poly(I:C)-induced upregulation of ACE2, IFNβ, and NF-κB signaling downstream TNFα and IL6 levels (Figures 4B–E). The inhibitory effects of Cepharanthine and Glucosamine were validated and shown in Figure 4F. Notably, solo use of Cepharanthine or Glucosamine inhibited viral RNA mimic poly(I:C)-induced upregulation of ACE2, however dual treatment of both drugs failed to further downregulate the level of ACE2. Consistently, similar results were observed in the regulation of IFNβ, TNFα and IL6 levels (Figures 4G–I). Furthermore, we found that Cepharanthine and Glucosamine treatment not only inhibited p-p65 and p-IκB levels, but also decreased ACE2 proteins levels (Figures 4J,K). These results suggested that clinical drugs Cepharanthine and Glucosamine not only inhibited ACE2 levels, but also suppressed the expression of IFNβ and NF-κB signaling.

In summary, through ISGs library screening, we found ACE2 levels were positively regulated by multiple ISGs. ACE2 levels were high correlated with ISGs-induced NF-κB levels. Furthermore, through clinical approved drugs screening, we found that clinical drugs Cepharanthine and Glucosamine significantly inhibited the expression of ACE2, IFNβ, TNFα and IL6, suggesting the possible protective role of those drugs in the prevention and clinical treatment of coronaviruses infection.

## Discussion

SARS-CoV-2 infection caused inflammatory cytokine storm, and subsequently induced severe organs damage and system disorders in such as lung, kidney, heart, intestines and central nervous system (Massad et al., 2020; Pesaresi et al., 2020; Rojas et al., 2020; Teng et al., 2020; Chu et al., 20212021; Komuro, 2021; Maccio et al., 2021). In this study, using human cell line BEAS-2B cell line and HMC3 cell line, we found that treatments of cytokine IFNβ, viral RNA mimic poly (I:C) and viral DNA mimic poly (dA:dT) significantly promoted the expression of ACE2, suggesting ACE2 was an ISG in human. This result was consistent with the reports from other groups (Zhuang et al., 2020; Ziegler et al., 2020). Viral infection caused the production of interferon and the subsequent ISGs in the infected cells (Kim et al., 1997; Levy et al., 2011; Garcia-Del-Barco et al., 2021). Meanwhile, ISGs played various diverse roles in maintaining immunologic homeostasis and controlling pathogens (Künzi and Pitha, 1996; Rödel et al., 2002; Schneider et al., 2014). Our previous study showed that several ISGs positively regulated interferon response (Cheng et al., 2016). However, the effects of these ISGs on ACE2 levels were not clear. Here, through an ISGs library screening, we found that multiple ISGs positively regulated ACE2 levels. Specifically, TNFR1 (ISG95) were found to promote the expression of ACE2 and IFNβ, and the activities of NF-κB signaling. These results further demonstrated that ISGs not only positively regulated interferon response, but also elevated the expression of ACE2 and enhanced the production of inflammatory cytokines.

Multiple evidences showed that increased ACE2 levels and inflammatory cytokine levels were presented in COVID-19 patients (Pinto et al., 2020; Soldo et al., 2020; Zhuang et al., 2020). However, the underlying mechanism were not clear. In this study, we found that ACE2 acted as an ISG, meanwhile its expression was also highly correlated with ISGs-induced NF-κB activities, but not the IFNβ levels. Moreover, we found that several ISGs were able to significantly promote both ACE2 expression and NF-κB activities. Consistently, it was reported that not only interferon treatment or viral infection increased ACE2 levels, several inflammatory cytokines stimulation, such as TNFα, IL6 and IL1β, also increased the level of ACE2 (Zhuang et al., 2020). These results indicated that individuals infected by other virus (such as influenza virus) or under the inflammatory conditions (such as inflammatory bowel disease, stroke or multiple sclerosis) were potentially more vulnerable to the infection of SARS‐CoV‐2, and were highly possible to develop inflammatory cytokine storm upon infection.

A growing clinical evidences suggested that cytokine storm contributed to the severity of COVID-19 infection, and acted as a critical cause of death of patients with COVID-19 (Fara et al., 2020; Soy et al., 2020). Therefore, we hypothesized that the drugs targeting both ACE2 and inflammatory cytokine levels might be effective in inhibiting the infection of coronaviruses and suppressing the resulting inflammatory cytokine storm. In the results, we found that treatments of Cepharanthine and Glucosamine significantly inhibited viral RNA mimic poly (I:C)-induced upregulation of ACE2, IL1β, and NF-κB downstream inflammatory cytokine TNFα and IL6. Cepharanthine was an alkaloid isolated from *Stephania cepharantha* Hayata that demonstrated anti-inflammatory, anti-oxidative, immune regulation and antiviral activities (Ershun et al., 2014; Matsuda et al., 2014; Aota et al., 2018; Rogosnitzky et al., 2020; Zhao et al., 2020). This drug was recently reported to produce inhibitory effects in the process of viral infection and replication of COVID-19 (Fan et al., 2020; Jeon et al., 2020). Glucosamine was a nature compound commonly found in our body, and produced beneficial effects to heart disease, diabetes and arthritis, through inhibition of oxidative stress and inflammatory activations (Hwang et al., 2013a; Hwang et al., 2013b; Shin et al., 2013; Park et al., 2016). Collectively, our findings suggested the possible protective effects of Cepharanthine and Glucosamine in clinical treatment of patients COVID-19.

In summary, in this study we domonstrated that ACE2 was an ISG in human cell lines. Its levels were highly correlated with ISGs-induced NF-κB activities. Moreover, through clinical approved drugs screening, we found Cepharanthine and Glucosamine were able to significantly inhibit viral RNA mimic poly (I:C)-induced the upregulation of ACE2, IL1β, and NF-κB downstream inflammatory cytokines, suggesting the potential application of these 2 drugs in the clinical treatment of COVID-19.

## Materials and Methods

### Cell Culture

BEAS-2B cell line, HMC3 cell line and HEK 293T cell line were obtained from the American Type Culture Collection and cultured in Dulbecco’s modified Eagle’s medium (DMEM; Gibco) containing 10% fetal bovine serum and 1% penicillin/streptomycin at 37°C in a humidified atmosphere with 5% CO_2_.

### Reagents

Poly (I:C) and Poly (dA:dT) were purchased from InvivoGen. IFNβ was purchased from Biolegend. The approved drug library was purchased from Topscience Co., Ltd.

ISG expression library was provided by Dr. Guangxia Gao (Institute of Biophysics, Chinese Academy of Science, China) and used in our previous study (Cheng et al., 2016).

### Luciferase Assay

Briefly, promoter regions from −1119 to 103 of human ACE2 was cloned into a pGL3-luciferase reporter vector. The sequence of plasmids was validated by sequencing. NF-κB luciferase reporter plasmid and IFNβ reporter luciferase plasmid were previously constructed and used in our previous studies (Cheng et al., 2016; Li et al., 2020). Luciferase activity were measured using Dual luciferase reporter assay system according to the manufacture’s protocol (Promega).

### Quantitative RT-PCR

Total RNA was extracted from indicated cell samples using Trizol reagent (Invitrogen, cat#15596018). 1 µg of RNA was used for the synthesize of cDNA using a one-step first strand cDNA synthesis kit (Transgen Biotech, cat#AT341). Quantitative real-time PCR was performed using 2 × SYBR Green PCR master mix (Transgen Biotech, cat#AQ131) and Agilent Mx3005P RT-PCR system. The expressions of tested genes were normalized to the expression of GAPDH, and the 2^−ΔΔCT^ method was used to analyze the relative changes in gene expression.

The primers for human ACE2, IFNβ, TNFα, IL6 and GAPDH were listed below:

Human ACE2: Forward: 5′-CGA​AGC​CGA​AGA​CCT​GTT​CTA-3′; Reverse: 5′-GGG​CAA​GTG​TGG​ACT​GTT​CC-3′;

Human IFN-β: Forward: 5′-ATG​ACC​AAC​AAG​TGT​CTC​CTC​C-3′; Reverse: 5′-GGA​ATC​CAA​GCA​AGT​TGT​AGC​TC-3′;

Human TNFα: Forward: 5′-CCT​CTC​TCT​AAT​CAG​CCC​TCT​G′; Reverse: 5′-GAG​GAC​CTG​GGA​GTA​GAT​GAG-3′;

Human IL6: Forward: 5′-ACT​CAC​CTC​TTC​AGA​ACG​AAT​TG′; Reverse: 5′-CCA​TCT​TTG​GAA​GGT​TCA​GGT​TG-3′;

Human GAPDH: Forward: 5′-GGA​GCG​AGA​TCC​CTC​CAA​AAT-3′; Reverse: 5′-GGC​TGT​TGT​CAT​ACT​TCT​CAT​GG3′;

### Western Blotting Analysis

Extracted proteins were separated by polyacrylamide gel electrophoresis (SDS-PAGE) and transferred to nitrocellulose membranes (GE Amersham, catalog no. 10600002), and incubated with the primary antibody for overnight at 4°C. Immunoblots were probed with the first antibody with anti-phospho-IκBα (Ser32) (#2859, Cell Signaling Technology), anti-IκBα (44D4) (#4814, Cell Signaling Technology), anti-phospho-p65 (Ser536) (#3033, Cell Signaling Technology), anti-p65 (D14E12) (#8242, Cell Signaling Technology), anti-ACE2 (#ab108209, Abcam) and *β*-actin (#CW0096M, CWBiotech). And ECL luminescent solution was used for detection.

### Statistical Analysis

Student’s t-test was used for comparisons between 2 groups. One-way analysis of variance (ANOVA) was used for multiple groups and correlation analysis. All statistical analysis were performed using GraphPad (Prism GraphPad Software). All values were expressed as the mean ± SEM. *p* value <0.05 were considered as significant.

## Data Availability

The original contributions presented in the study are included in the article/Supplementary Material, further inquiries can be directed to the corresponding author.
